# Dataset on performance of solar powered agricultural produce cooling storage system under tropical conditions

**DOI:** 10.1016/j.dib.2019.104649

**Published:** 2019-10-13

**Authors:** S.O. Oyedepo, J.A. Omoleye, O. Kilanko, C.S. Ejike, B.F. Bolarinwa–Odunayo, U. Idemili, G. Odewole

**Affiliations:** a Department of Mechanical Engineering, Covenant University, Nigeria; b Department of Chemical Engineering, Covenant University, Nigeria

**Keywords:** Ambient temperature, Relative humidity, Evaporative cooling system, Wet bulb temperature, Cooling chamber

## Abstract

Much of the post-harvest loss of agricultural produce in developing countries is due to lack of proper storage facilities. Agricultural produce such as peppers, tomatoes and fruits are highly perishable in nature; thus, maintaining the optimal air conditions inside the storage cabinet helps extending their shelf lives. The datasets contained in this paper are performance test carried out on agricultural produce cooling storage system under tropical conditions for various cooling pads (jute fibre) thickness and under no load and load conditions. The parameters recorded under these conditions include wet and dry bulb temperatures, relative humidity of the surroundings, relative humidity and temperature within the cooling chamber taken consecutively for 4 days with different pad thickness and for 5 days and 8days under no load and load conditions, respectively. Results obtained show that pad thickness of 80 mm has the highest cooling efficiency of 84.7% and temperature within the cooling chamber was found dropped to 25 °C and average relative humidity of cooling chamber increased to 82.4% as compared to 64.8% for ambient condition.

**Table:** Specifications Table

Subject Area	Engineering
More Specific Subject area	Mechanical Engineering, Heat transfer and Thermo- fluid Engineering
Type of Data	Tables, Figures and graphs
How Data was Acquired	The internal temperature and humidity of cooling chamber were measured using Floureon RC-4HC Data logger device. The data logger was set to record data at every 30 minutes through a period of 8 hours for 8 days. RC-4HC temperature and humidity data logger when connected to computer, the software reads data automatically, and form reports. It has LCD screen which display the temperature, time, over temperature alarm, temperature upper/lower limit and maximum/minimum temperature. There are two sensors – internal and external used to record temperature over a long time. Temperature measuring range from -30 °C to +60 °C with accuracy of ±0.6 °C. Humidity range from 0 to 99% RH with accuracy of ±3% RH. Record capacity is RC-4HC 16000 points (MAX) while record interval is from 10 s to 24 h adjustable. The wet bulb and dry bulb temperatures of the surrounding were measured using wet bulb and dry bulb hygrometer alongside with psychometric chart. Digital anemometer was used to determine the air velocity entering into system.
Data Format	Raw and analysed
Experimental Factor	Performance of the cooling storage system was determined from wet bulb and dry bulb temperatures readings taken under no load and load conditions with different pad thickness varied from 20 mm to 80 mm.
Experimental Feature	Tests were first carried out on different cooling pad thicknesses ranged from 20 mm to 80 mm purposely to determine which thickness gives highest cooling efficiency. After which, load and no load tests were carried out on the designed post harvested agricultural produce cooling storage system at every 30 minutes through a period of 8 hours for 8 days.
Data source location	Department of Mechanical Engineering, Covenant University, Ota, Nigeria
Data Accessibility	Data are available within this article

**Table:** 

**Value of the Data** • The given dataset can be used to provide information on the storage temperature, humidity requirements and the length of time agricultural produce can be kept without a decline in market value. • The cooling efficiency calculated can be used for benchmarking performance of such cooling storage in another location under similar condition. • The given dataset can be used to assess the impact of the cooling system on the stored agricultural produce. • The given data will show researchers in the field of energy management and sustainable development the potential of solar PV to power cooling storage system especially in developing countries where access to electricity is limited.

## 1 Data

The temperatures and humidity of cooling chamber and surrounding were collected and a set of experimental data was generated. Tests were first carried out on different cooling pad thicknesses ranged from 20 mm to 80 mm purposely to determine which thickness gives highest cooling efficiency (Table 1, Table 2, Table 3, Table 4). After which, no load and load tests were carried out on the designed post harvested agricultural produce cooling storage system at every 30 minutes through a period of 8 hours for 5 days (no load tests) and 8 days (load tests).

**Table 1:** Experimental Data for cooling pad thickness of 20 mm.

Time (Mins.)	T_C_ (°C)	R.H_C_ (%)	DBT (°C)	WBT (°C)	R.H_A_ (%)
0	35.8	63.7	35	29	63.5
30	29.7	78	33	28	68.5
60	30.2	77.7	34	28	63
90	30.2	77.2	33	27	62.5
120	29.8	78	34	27	58
150	29.8	78.5	35	28	58.5
180	30.2	78.1	33	27	62.5
210	30	78.1	33	28	68.5
240	30	78.5	33	28	68.5
270	29.9	76.7	34	29	69
300	30	76.3	33	27	62.5
330	29.8	78.3	36	28	54
360	28.6	79.5	34	28	63
390	28.2	80.3	33	26	57
420	28	81.5	31	27	74.5
450	27.7	82.3	31	27	74.5
480	27.3	83.1	29	26	78.5

**Key:** T_c_ – Temperature of cooling chamber, R.H_C_ – Relative humidity of cooling chamber, DBT – Dry bulb temperature, WBT – Wet bulb temperature, R.HA – Relative humidity of the surroundings.

**Table 2:** Experimental data for cooling pad thickness of 40 mm.

Time (Mins.)	T_C_ (°C)	R.H_C_ (%)	DBT (°C)	WBT (°C)	R.H_A_ (%)
0	34.9	63	35	28	58.5
30	29.5	76.2	35	27	53.5
60	29	78.3	34	27	58
90	29.4	77.5	36	28	54
120	29.8	77.6	34	27	58
150	29.9	78.5	37	28	50.5
180	29.8	77.1	39	29	47.5
210	30.3	75.9	38	29	51
240	30.8	74.2	35	28	58.5
270	31.1	74.7	37	29	55
300	29.7	76.7	35	28	58.5
330	29.3	77	34	27	58
360	28.5	78.2	33	26	57
390	28.3	79.5	33	26	57
420	28.1	80.3	33	27	62.5
450	27.6	81	31	26	67.5
480	27.2	82.2	30	26	73

**Table 3:** Experimental data for cooling pad thickness of 60 mm.

Time (Mins.)	T_C_ (°C)	R.H_C_ (%)	DBT (°C)	WBT (°C)	R.H_A_ (%)
0	31.7	66.4	31	26	61.5
30	29.4	80.1	31	27	73.5
60	29.2	81.4	32	27	68
90	29.1	82.2	34	28	63
120	28.9	82.6	34	28	63
150	29.2	83.1	39	30	51.5
180	29.2	83.5	39	30	51.5
210	29.4	83.5	40	30	48
240	29.5	83.6	38	30	56
270	29.3	83.5	36	29	59
300	28.9	84.3	33	27	63.5
330	28.6	84.2	32	27	68
360	28.2	83.7	31	27	73.5
390	28	84.1	31	26	67.5
420	27.7	84.8	29	26	78.5
450	27.4	84.8	29	26	78.5
480	27.1	85.5	28	25	78

**Table 4:** Experimental data for cooling pad thickness of 80 mm.

Time (30 mins)	T_C_ (°C)	RH_C_ (%)	DBT (^o^C)	WBT (°C)	RH_A_ (%)
0	30.4	64.3	30	29	63
30	25.4	79.3	32	29	70
60	26.2	80.7	32	28	74
90	26.8	80.4	33	28	68.5
120	27.6	79.3	34	27	58
150	28.1	76.8	30	26	73
180	27.9	78.1	34	26	53
210	27.7	78.9	32	27	68
240	27.2	80.1	33	27	62.5
270	27	80	33	26	57
300	26.6	80.4	36	27	50
330	26.3	80.3	33	27	62.5
360	26.2	80.7	30	26	73
390	27	80	29	25	66
420	27.2	80.1	29	26	78.5
450	27.6	82.3	27	25	75
480	28.1	86.8	28	25	78
Average	27.3	79.3	31.5	26.7	66.5

## 2 Experimental design, materials and methods

**Fig. 1:**
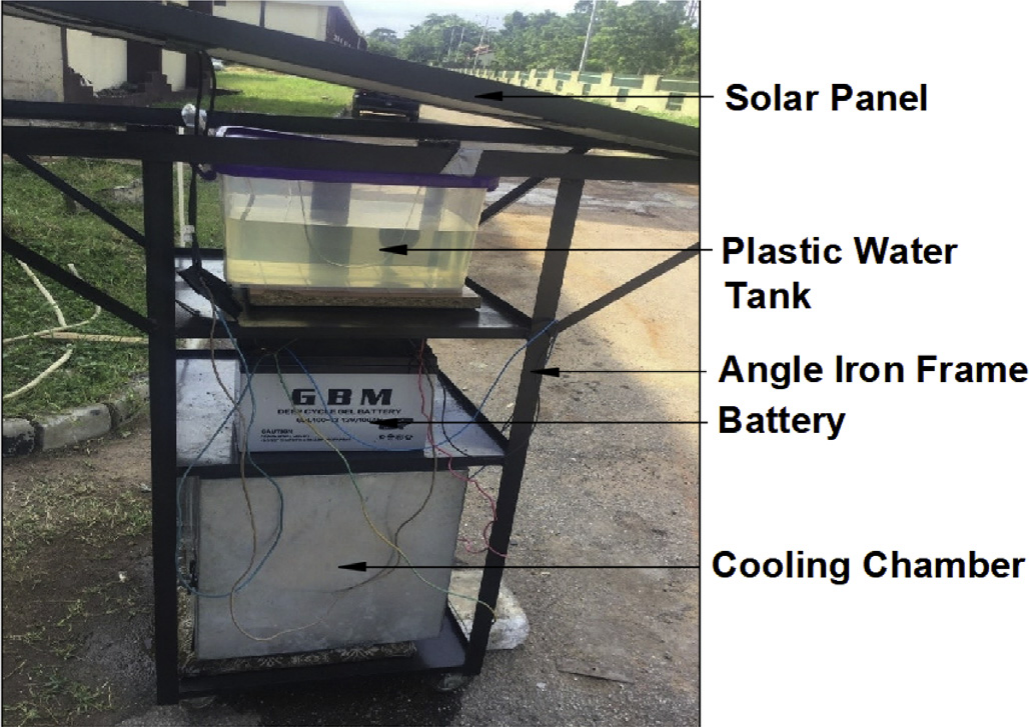
Solar powered vegetable & fruit storage system.

A 500 mm × 500 mm × 500 mm (Length x depth x width) postharvest agricultural produce cooling storage system was designed and constructed as shown in Fig. 1. The cooling system basically consists of a cubical shaped storage chamber, a cooling fan, and a cooling pad. The cubical shaped cooling chamber was of dimension 0.125 m³ made of aluminum on the inside and galvanized mild steel on the outside, it is internally insulated with polystyrene to prevent heat exchange with the environment, a cooling fan and a porous cooling pad made from jute fiber and a submersible pump of 50 W power rating having a flow rate of 240l/hr and a maximum static head of 3 m. A water reservoir of capacity 20 l, at the top of the cooling system transferring water being pumped through a piping system to the cooling pad to keep it continually wet. The system relies solely on the concept of cooling by evaporation [[1], [2], [3]], when the system is set up and is under operation, the dry air from the cooling fan goes directly to the wet surface of the cooling pad and evaporates the water present in the pad, thus drawing energy from its surroundings to produce cooling effect into the cooling chamber [4,5].

In this study, Floureon RC- 4HC Data logger device was used measure the internal temperature and humidity of cooling chamber. The data logger was set to record data at every 30 minutes through a period readings were taken. Floureon RC-4HC temperature and humidity data logger when connected to computer, the software reads data automatically, and form reports. It has LCD screen which display the temperature, time, temperature upper/lower limit and maximum/minimum temperature. There are two sensors – internal and external used to record temperature over a long time. Temperature measuring range from −30 °C to +60 °C with accuracy of ±0.6 °C. Humidity range from 0 to 99% RH with accuracy of ±3%RH. Record capacity is RC-4HC 16000 points (MAX) while record interval is from 10 s to 24hours adjustable. The wet bulb and dry bulb temperatures of the surrounding were measured using wet bulb and dry bulb hygrometer alongside with psychometric chart. Digital anemometer was used to determine the air velocity entering into system.

Cooling efficiency of the cooling storage system was calculated using different jute fiber pads of different thicknesses based on the established formula given as [6,7]:

(1) $$\eta =\frac{T_{db}-T_{c}}{T_{db}-T_{wb}}\times 100$$

where:

T_db_ = ambient air dry bulb temperature in °C.

T_wb_ = ambient air wet bulb temperature in °C.

T_c_ = dry bulb temperature of the cooler in °C.

The computed cooling efficiencies under no load condition for 20 mm, 40 mm, 60 mm and 80 mm cooling pads are 61.4%; 72.2%; 82.4% and 84.7%, respectively.

**Fig. 2:**
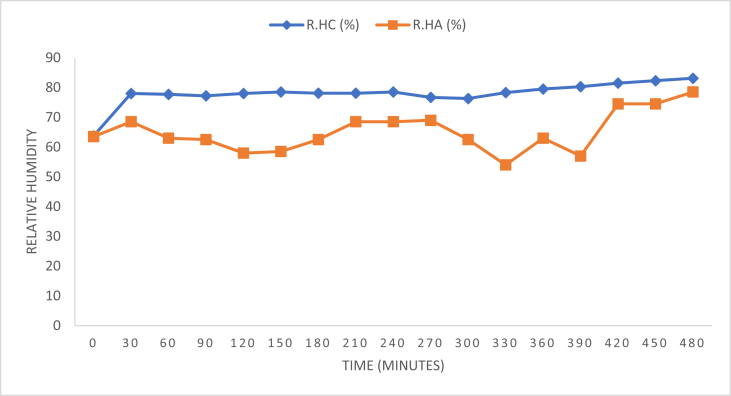
Relative humidity readings for pad thickness of 80 mm.

**Fig. 3:**
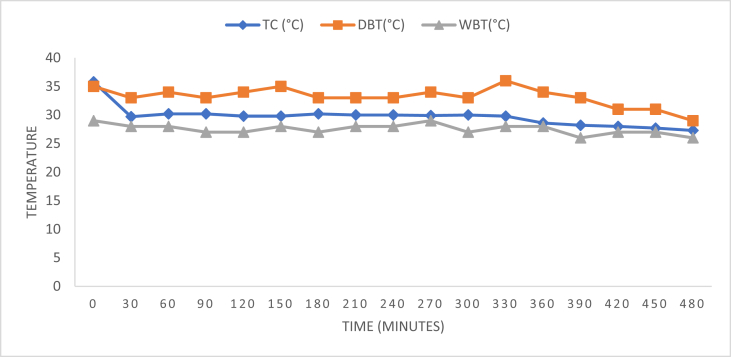
Temperature readings for pad thickness 80 mm.

Analysis displaying the data for a cooling pad of thickness 80 mm are shown in Fig. 2, Fig. 3.

Cooling pad of thickness 80 mm was used to examine performance of the storage cooling system under no load and load conditions for 5 days and 8 days, respectively (Table 5, Table 6, Table 7, Table 8, Table 9, Table 10, Table 11, Table 12, Table 13, Table 14, Table 15, Table 16, Table 17).

**Table 5:** Experimental data for cooling storage under no load condition (Day 1).

Time (30 mins)	T_C_ (°C)	RH_C_ (%)	DBT (^o^C)	WBT (°C)	RH_A_ (%)
0	34.1	63.9	34	27	58
30	27	73.3	34	26	53
60	28	75.9	35	27	53.5
90	26.6	75.9	34	26	53
120	27.7	75.1	34	27	58
150	26.4	75	33	26	57
180	27.6	77	34	27	58
210	26.2	73.9	34	26	53
240	25.9	72.6	33	25	52
270	26.4	72.7	34	26	53
300	27.1	70.5	35	26	49
330	27	76	35	26	49
360	27.8	77	33	27	62.5
390	26.6	77.2	33	26	57
420	26.9	77.6	32	26	62
450	25.9	77.6	32	25	56
480	25.8	75.9	31	25	55.5

**Table 6:** Experimental data for cooling storage under No load condition (Day 2).

Time (Mins.)	T_C_ (°C)	R.H_C_ (%)	DBT (°C)	WBT (°C)	R.H_A_ (%)
0	35.8	64.5	34	31	63.5
30	29.7	71.8	33	30	68.5
60	30.2	73.1	33	30	63
90	30.2	72.2	32	29	62.5
120	29.8	72.2	33	29	74.1
150	29.8	73.0	34	32	58.5
180	30.2	73.5	34	31	62.5
210	30	74.1	34	31	68.5
240	30	74.7	32	30	68.5
270	29.9	75.1	32	28	69
300	30	75.6	32	29	62.5
330	29.8	76	30	27	54
360	28.6	76	31	27	63
390	28.2	76.3	30	27	57
420	28	76.8	30	28	74.5
450	27.7	76.8	29	27	74.5
480	27.3	77.2	29	26	78.5
Average	29.7	74.3	31	28	74.1

**Table 7:** Experimental data for cooling storage under No load condition (Day 3).

Time (Mins.)	T_C_ (°C)	R.H_C_ (%)	DBT (°C)	WBT (°C)	R.H_A_ (%)
0	32.6	69.9	33	28	68.5
30	29.2	80.5	34	28	63
60	29.1	81.8	33	27	62.5
90	28.8	81.7	34	28	63
120	27.8	79.3	33	27	62.5
150	26.1	81.6	31	26	67.5
180	25.7	83.6	30	25	67
210	25.5	84.4	27	24	77.5
240	25.3	84.7	27	24	77.5
270	25.2	85.1	27	24	77.5
300	25	85.1	28	25	78
330	25	85.5	27	24	77.5
360	24.9	85.4	27	24	77.5
390	24.8	85.9	27	24	77.5
420	24.8	85.8	27	24	77.5
450	24.7	85.8	27	24	77.5
480	24.7	86.8	26	24	85
Average	26.4	83.1	27.8	25.3	72.8

**Table 8:** Experimental data for cooling storage under No load condition (Day 4).

Time (30 mins)	T_C_ (°C)	RH_C_ (%)	DBT (^o^C)	WBT (°C)	RH_A_ (%)
0	34.9	63	35	28	58.5
30	29.5	76.2	35	27	53.5
60	29	78.3	34	27	58
90	29.4	77.5	36	28	54
120	29.8	77.6	34	27	58
150	29.9	78.5	37	28	50.5
180	29.8	77.1	39	29	47.5
210	30.3	75.9	38	29	51
240	30.8	74.2	35	28	58.5
270	31.1	74.7	37	29	55
300	29.7	76.7	35	28	58.5
330	29.3	77	34	27	58
360	28.5	78.2	33	26	57
390	28.3	79.5	33	26	57
420	28.1	80.3	33	27	62.5
450	27.6	81	31	26	67.5
480	27.2	82.2	30	26	73

**Table 9:** Experimental data for cooling storage under No load condition (Day 5).

Time (30 mins)	T_C_ (°C)	RH_C_ (%)	DBT (^o^C)	WBT (°C)	RH_A_ (%)
0	35.8	63.7	35	29	63.5
30	29.7	78	33	28	68.5
60	30.2	77.7	34	28	63
90	30.2	77.2	33	27	62.5
120	29.8	78	34	27	58
150	29.8	78.5	35	28	58.5
180	30.2	78.1	33	27	62.5
210	30	78.1	33	28	68.5
240	30	78.5	33	28	68.5
270	29.9	76.7	34	29	69
300	30	76.3	33	27	62.5
330	29.8	78.3	36	28	54
360	28.6	79.5	34	28	63
390	28.2	80.3	33	26	57
420	28	81.5	31	27	74.5
450	27.7	82.3	31	27	74.5
480	27.3	83.1	29	26	78.5

**Table 10:** Experimental data for cooling storage under load condition (Day 1).

Time (30 mins)	T_C_ (°C)	RH_C_ (%)	DBT (^o^C)	WBT (°C)	RH_A_ (%)
0	33.6	58.3	32	30	58
30	31.1	67.3	32	30	53
60	31	69.1	33	31	53.5
90	30.5	68.1	31	30	53
120	30.4	70	32	30	58
150	31.2	69.7	31	29	57
180	30.3	71.3	31	29	56
210	29.6	73.5	30	28	53
240	29.2	73.9	30	29	52
270	28.8	75.6	30	28	53
300	28.6	79.1	29	28	49
330	28.4	80.8	29	27	49
360	28.3	81.4	29	28	62.5
390	28.3	81.9	29	28	57
420	28.2	82.6	28	29	62
450	28.0	83.4	28	27	56
480	27.7	83.8	28	27	55.5

**Table 11:** Experimental data for cooling storage under load condition (Day 2).

Time (Mins.)	T_C_ (°C)	R.H_C_ (%)	DBT (°C)	WBT (°C)	R.H_A_ (%)
0	32	69.3	33	31	68.5
30	31.6	73.6	34	31	63
60	30.1	74.1	33	31	62.5
90	29.8	73.2	33	30	63
120	29.8	74.1	32	29	62.5
150	29.5	74.6	31	28	67.5
180	29.1	78	30	27	67
210	29.1	79.3	28	27	77.5
240	28.7	80.1	28	26	77.5
270	28.6	80.9	28	26	77.5
300	28	81.7	29	27	78
330	27.6	82	28	28	77.5
360	27.2	82.5	28	27	77.5
390	26.9	83.3	28	27	77.5
420	26.5	85	28	26	77.5
450	26	85.6	28	25	77.5
480	25.8	86	27	25	79

**Table 12:** Experimental data for cooling storage under load condition (Day 3).

Time (Mins.)	T_C_ (°C)	R.H_C_ (%)	DBT (°C)	WBT (°C)	R.H_A_ (%)
0	31.8	65.5	30	27	61.5
30	27.5	74	30	26	73.5
60	26.5	76	31	26	68
90	25.9	78.1	29	26	63
120	25.5	81	29	25	63
150	25.3	82.7	28	25	51.5
180	25.4	83.1	28	25	51.5
210	25.5	84	28	25	48
240	25.6	85.2	27	25	56
270	25.5	86	29	26	59
300	25.5	86.4	28	25	63.5
330	25.4	86.8	29	26	68
360	25.4	87.8	29	25	73.5
390	25.4	87.2	28	24	67.5
420	25.6	87.6	28	24	78.5
450	25.7	87.6	28	25	78.5
480	25.5	87.2	27	24	78

**Table 13:** Experimental data for cooling storage under load condition (Day 4).

Time (Mins.)	T_C_ (°C)	R.H_C_ (%)	DBT (°C)	WBT (°C)	R.H_A_ (%)
0	30.5	74.1	33	28	65
30	27.1	80.5	31	27	72.4
60	27.2	82.2	32	27	64
90	27.4	82.2	30	27	69.6
120	27.3	82.3	30	26	61.2
150	27.4	82.7	29	26	52.4
180	27.2	83.9	29	26	52.4
210	27	83.9	29	26	50
240	26.7	84.7	28	26	55
270	26.2	85	30	27	58
300	25.9	85.7	29	26	64.1
330	25.6	86.1	30	27	68
360	25.6	86.4	30	26	72.5
390	25.3	86.7	30	25	66
420	25.2	87	29	25	79
450	25	87.2	29	26	74
480	24.8	87.6	28	25	73.2

**Table 14:** Experimental data for cooling storage under load condition (Day 5).

Time (Mins.)	T_C_ (°C)	R.H_C_ (%)	DBT (°C)	WBT (°C)	R.H_A_ (%)
0	31.3	70.2	33	27	93
30	28.4	72	31	26	80
60	28	75.6	32	26	74
90	27.2	76.1	30	26	68.5
120	27.1	76.7	30	25	58
150	26.7	78.6	29	26	73
180	26.2	80.4	29	25	53
210	25.8	82.1	29	25	68
240	25.6	84.6	28	25	62.5
270	25.5	86.2	30	26	57
300	25.2	87.5	29	25	50
330	25.1	87.5	29	26	62.5
360	25	87.5	29	25	73
390	24.9	87.9	28	24	66
420	24.8	88.2	28	24	78.5
450	24.7	88.2	28	25	85
480	24.7	88.2	27	24	78

**Table 15:** Experimental data for cooling storage under load condition (Day 6).

Time (30 mins)	T_C_ (°C)	RH_C_ (%)	DBT (^o^C)	WBT (°C)	RH_A_ (%)
0	30.8	63.9	33	31	63
30	29	73.3	32	30	67
60	29	75.9	31	29	68
90	28.6	75.9	31	29	72
120	28.4	76	30	28	56
150	28.2	78	30	28	56
180	28	78	31	28	64
210	27.5	79	29	27	68
240	27.4	80	29	27	56
270	26.9	80.5	30	26	68
300	26.3	81.3	29	26	52
330	26.4	87.5	31	26	68
360	26.4	87.5	30	25	73.5
390	26.1	87.9	29	24	67.5
420	25.9	88.2	29	24	78.5
450	25.5	88.2	27	25	78.5
480	25.5	88.2	28	24	78

**Table 16:** Experimental data for cooling storage under load condition (Day 7).

Time (Mins.)	T_C_ (°C)	R.H_C_ (%)	DBT (°C)	WBT (°C)	R.H_A_ (%)
0	33.6	81	29	28	58.3
30	31.1	80	31	28	67.3
60	31	73	31	27	69.1
90	30.2	72.2	32	27	68.5
120	29.8	72.2	33	26	57.2
150	29.8	73.0	29	25	72.2
180	28.3	77.1	34	27	58
210	27	84.3	33	27	62.6
240	26.8	84.6	34	28	63.2
270	26.7	85.5	33	27	62.6
300	26.5	85.4	32	27	58
330	26.4	85.9	31	27	73.2
360	26.4	86.4	30	26	73
390	26.1	86.8	29	25	66
420	25.9	87.2	29	26	78.5
450	25.6	87.3	27	25	85
480	25.5	87.3	28	25	78

**Table 17:** Experimental data for cooling storage under load condition (Day 8).

Time (30 mins)	T_C_ (°C)	RH_C_ (%)	DBT (^o^C)	WBT (°C)	RH_A_ (%)
0	32.8	86	32	30	65.2
30	30.8	77.6	32	28	73.7
60	29.7	80.4	32	27	67.7
90	29.6	80.7	33	27	62.6
120	29	79.3	33	28	68.3
150	28.6	80	32	29	76.8
180	28.3	78.1	33	29	74.1
210	28.1	79.5	31	28	62.6
240	27.8	80.1	30	27	63.2
270	27.7	80.2	30	27	62.6
300	27.1	80.4	29	25	72
330	27	80.8	30	26	72.7
360	26.5	81.2	29	26	78
390	26.2	82.4	29	25	72.7
420	25.8	83.7	29	26	78
450	25.5	87.3	27	25	78.5
480	24.6	87.3	28	24	78

Table 18 presents computed cooling efficiency of the storage cooling system for the period of 8 days under loading condition. The highest cooling efficiency (91.1%) was recorded on the 5th day of the experiment (see Table 18).

**Table 18:** Computed cooling efficiency under load condition.

Days	Cooling Efficiency (%)
1	47.06
2	55
3	72.7
4	88.5
5	91.1
6	50
7	62.2
8	67.7

**Fig. 4:**
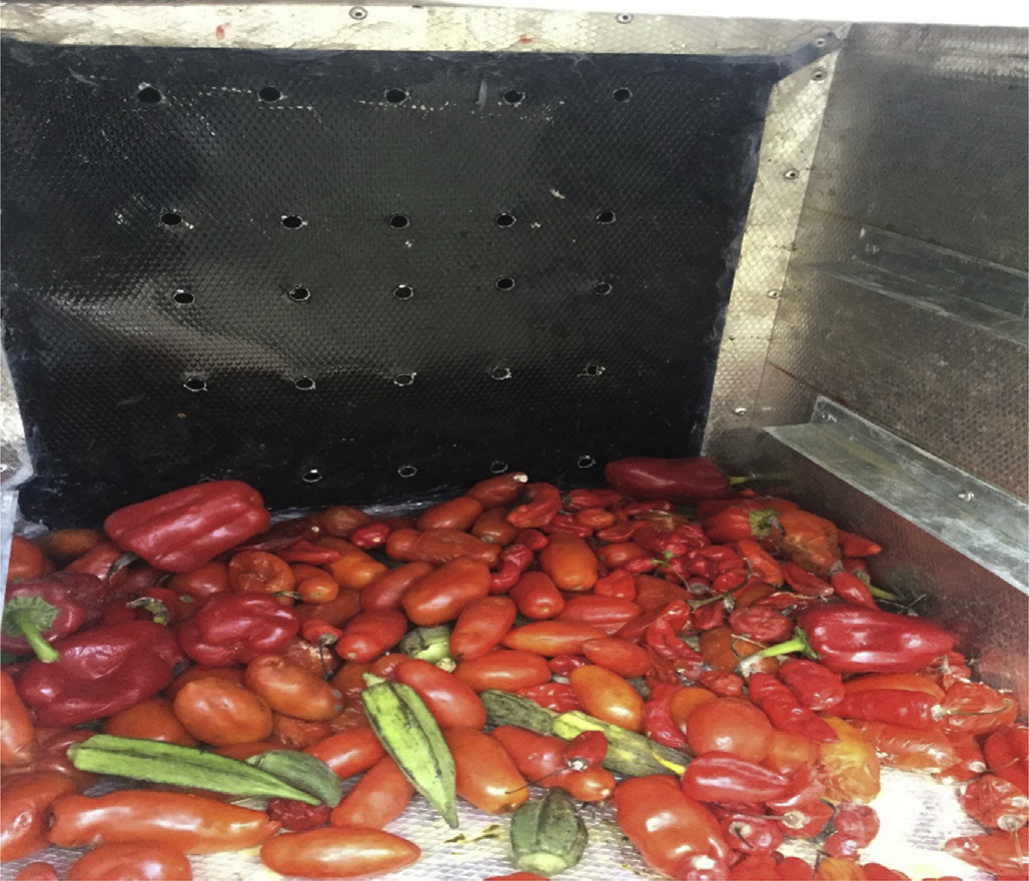
Loaded Agricultural Produce Postharvest Storage System showing the experimental set up.

Fig. 4 shows loaded agricultural postharvest storage cooling system after 8 days during the experiment.

From this study, it was observed that:

➢ The drop in temperature of cooling cabinet of the storage system was relatively high during hot and sunny conditions and relatively low smaller under cloudy and rainy conditions.
➢ Increase in air flow rate enhances the rate of evaporation efficiently.
➢ The agricultural produce (tomatoes, okra and pepper) kept in cooling chamber of agricultural cooling storage system maintained their freshness and firmness after 8 days in comparison to those kept under ambient air conditions.

## References

[1] Liberty J.T., Ugwuishiwua B.O., Pukumab S.A., Odoc C.E. (2013). Principles and application of evaporative cooling systems for fruits and vegetables preservation. Int. J. Curr. Eng. Technol..

[2] Kulkarni R.K., Rajput S.P.S., Gutte S.A., Patil D.M. (2014). Laboratory performance of evaporative cooler using jute fiber ropes as cooling media. Int. J. Eng. Res. Appl..

[3] Rodrigo C.M., Lorenzo T.D., Sebastinne M.S.M., Irish K.A.O., Neissa A.Q., Dustin J.R.S. (2017). Automated electronic evaporative cooler for fruits and vegetables preservation. Int. J. Eng. Sci. Res. Technol..

[4] Zakari M.D., Abubakar Y.S., Muhammad Y.B., Shanono N.J., Nasidi N.M., Abubakar M.S., Muhammad A.I., Lawan I., Ahmad R.K. (2016). Design and construction of an evaporative cooling system for the storage of fresh tomato. ARPN J. Eng. Appl. Sci..

[5] Ayomide O.B., Ajayi O.O., Banjo S.O., Ajayi A.A. (2017). Data on the no-load performance analysis of a tomato postharvest storage system. Data in Brief.

[6] Babalola P.O., Bolu C.A., Inegbenebor A.O., Oyedepo S.O., Kilanko O., Adeyemi G.A. (2019). Application of solar photovoltaic system to power air blower and mixing mechanism in a tilting furnace. World Rev. Sci. Technol. Sustain. Dev..

[7] Al-suleiman F. (2002). Evaluation of performance of local fibers in evaporative cooling. Energy Convers. Manag..

